# Artesunate Restrains Maturation of Dendritic Cells and Ameliorates Heart Transplantation-Induced Acute Rejection in Mice through the PERK/ATF4/CHOP Signaling Pathway

**DOI:** 10.1155/2021/2481907

**Published:** 2021-08-21

**Authors:** Yuanyang Chen, Sihao Zheng, Zhiwei Wang, Xin Cai, Yanjia Che, Qi Wu, Shun Yuan, Xiaohan Zhong

**Affiliations:** ^1^Department of Cardiovascular Surgery, Renmin Hospital of Wuhan University, No. 238 Jiefang Road, Wuhan, 430060 Hubei, China; ^2^Cardiovascular Surgery Laboratory, Renmin Hospital of Wuhan University, 9# Zhangzhidong Road, Wuhan, 430000 Hubei Province, China

## Abstract

**Background:**

Heart transplantation (HT) is the only effective treatment for end-stage heart failure because it can effectively improve the survival rate and quality of life of patients with heart failure. Artesunate (ART) is an artemisinin derivative, with good water solubility and higher oral bioavailability. The main aim of this study was to determine the role of ART in HT mice.

**Methods:**

In animal experiments, mice were divided into the control group, HT group, low ART+HT group, and high ART+HT group. Next, inflammatory cell infiltration, oxidative stress injury, and myocardial cell apoptosis were determined in heart tissue. The proportion of multiple lymphocytes in spleen and lymph nodes was then determined using flow cytometry. In addition, cell experiments were conducted to determine the changes in expression of surface maturation markers of BMDC and changes in intracellular reactive oxygen species after LPS stimulation. Finally, western blot analysis was performed to determine the levels of endoplasmic reticulum stress-related proteins (CHOP/ATF4/PERK).

**Results:**

The survival time of mice in the ART treatment group was significantly prolonged and was positively correlated with the dose. In animal experiments, ART significantly reduced inflammatory cell infiltration in heart tissue and the proportion of CD4+CD8+ T cells in spleens and lymph nodes. Moreover, ART treatment lowered the 8-OHdg in hearts and myocardial apoptosis. In cell experiments, ART treatment slowed down the development and maturation of BMDCs by inhibiting the expression of endoplasmic reticulum stress-related proteins. Furthermore, the treatment alleviated the oxidative stress damage of BMDCs.

**Conclusion:**

ART can inhibit maturation of dendritic cells through the endoplasmic reticulum stress signaling pathway, thereby alleviating acute rejection in mice after heart transplantation.

## 1. Introduction

Heart failure (HF) is the final stage in the development of heart diseases. A recent epidemiological survey reported that the prevalence of heart failure among Chinese residents≧35 years of age is 1.3% [1]. Notably, heart transplantation is the only effective treatment for end-stage heart failure because it can effectively improve the survival rate and quality of life of patients with heart failure. However, in addition to the limited number of donor hearts, the development of heart transplantation is affected by many complications. Short-term complications include graft dysfunction, rejection, and various infections. On the other hand, the long-term survival of patients is significantly affected by the occurrence of malignancy and cardiac allograft vasculopathy (CAV) [2]. It is worth noting that the survival rate of patients after heart transplantation is mainly limited by immune rejection. Both acute and chronic rejections are important factors that destroy the function of the graft, which greatly restricts the development of heart transplantation [3, 4]. Therefore, this calls for urgent studies on how to alleviate the rejection of a heart transplant.

Evidence suggests that T cells and monocyte/macrophage lineage cells modulate acute rejection [5]. Dendritic cells (DCs), the most prominent antigen-presenting cells (APC), regulate initial and adaptive immune responses and are ideally positioned to serve a priming and central role in the immune response. The ability of DCs to activate immune response or induce immune tolerance is closely associated with their maturation status. Mature DCs (mDCs) express high levels of major histocompatibility complex-II (MHC-II) and costimulatory molecules (CD40, CD80, and CD86) on their surface, which can promote T cell activation and accelerate transplant rejection [6]. Moreover, heart transplantation leads to a large accumulation of reactive oxygen species (ROS). A previous study reported that ROS can enhance endoplasmic reticulum (ER) stress to regulate DC homeostasis, thereby regulating the immune-mediated tumor killing [7]. Notably, endoplasmic reticulum stress is mainly regulated by three signaling pathways, including PRK-like ER kinase (PERK), activated transcript factor 6 (ATF6), and inositol requiring enzyme 1 (IRE1). Among the three, the PERK/eukaryotic initiation factor 2*α* (eIF2*α*) pathway is the most closely associated with oxidative stress. It has been reported that exogenous hydrogen peroxide (H_2_O_2_) or organic oxidants, such as tert-butyl hydroperoxide, menadione, or diamide, can cause phosphorylation of eIF2*α*, induce activated transcript factor 4 (ATF4), and integrate stress response [8].

In recent years, researchers have focused on the therapeutic potential of natural plant products. Among the nature-derived phytochemicals, artemisinin and its derivative (artesunate) have received increasing attention due to their great biological effects. Artesunate (ART) is mainly derived from *Artemisia annua* which has high edible and medicinal value. *Artemisia annua* is a plant of the genus Compositae, which is mainly distributed in China, North Korea, Japan, Vietnam, Myanmar, India, and Nepal. ART has good water solubility and higher oral bioavailability because of its hemisuccinate groups. In addition to antimalarial effects, ART has anti-inflammatory and anti-immune functions. Studies have shown that ART participates in oxidative stress and DNA damage and repair (base excision repair, homologous recombination, and nonhomologous end connection) [9–11]. Moreover, ART can regulate a variety of cell death (apoptosis, autophagy, iron hyperplasia, and necrosis) through regulating several signal transduction pathways (for example, the Wnt/*β*-catenin pathway and the AMPK pathway) [12, 13]. A previous study suggested that ER stress-derived damage-associated molecular patterns (DAMPs) activate DCs, which are then capable of polarizing naïve T cells [14]. This study hypothesized that ART can inhibit the oxidative stress caused by ischemia-reperfusion (IR) and inhibit the development and function of DCs through the ER pathway in the heart transplantation model. Furthermore, ART can reduce the incidence of immune rejection after heart transplantation.

## 2. Materials and Methods

### 2.1. Animals and Ethics Statement

Male BALB/c and C57 mice (average weight: 25 g; age: 6-8 weeks) were purchased from the Animal Center of Hubei Disease Control and were allowed to acclimate to lab conditions for one week prior to commencement of the experiments. The experimental scheme was approved by the Ethics Committee of Wuhan University. Handling of all animals was performed in accordance with the Wuhan Directive for Animal Research and Current Guidelines for the Care and Use of Laboratory Animals published by the National Institutes of Health. Animal experiments passed the ethical review of laboratory animal welfare of the Renmin Hospital of Wuhan University (IACUC Issue No.20201107). In addition, all experiments involving animals were performed in the Animal Experiment Center of Renmin Hospital of Wuhan University.

### 2.2. Reagents

Fluorochrome-conjugated antibodies for mouse CD4 (FITC), CD8*α* (PE), CD25 (PE), FOXP3 (APC), CD11c (PE), CD86 (FITC), and MHC-II (FITC) were purchased from MultiSciences Biotechnology (Hangzhou, China). Immunoblotting antibodies for CD4, CD8, FOXP3, CD11c, and MHC-II were purchased from Becton, Dickinson and Company (New Jersey, US). TUNEL staining kit, ROS test kit, and lipopolysaccharides (LPS) were purchased from Servicebio (Wuhan, China), while artesunate was purchased from Yuanye Biology (Shanghai, China). CCT020312 and mouse recombinant macrophage colony-stimulating factor (M-CSF) were purchased from MedChemExpress (China). Moreover, western blotting antibodies for GAPDH, total-PERK, p-PERK, ATF4, and CHOP were purchased from Cell Signaling Technology (Massachusetts, US).

### 2.3. Animal Groups and Drug Treatment

Mice were randomly assigned to the following four groups: normal control group (NC, *n* = 6); heart transplantation group (HT, *n* = 6); heart transplantation with low ART group (LART, *n* = 6, 10 mg/kg), and heart transplantation with high ART group (HART, *n* = 6, 50 mg/kg). The NC group was composed of normal C57 mice, while the HT group was a positive control group with BALB/c mice as donors and C57 mice as recipients. In the LART and HART groups, 10 mg/kg and 40 mg/kg ART were used to pretreat the receptor five days before HT and treat the receptor seven days after HT, respectively.

### 2.4. Culture of Bone Marrow-Derived DCs (BMDCs)

BMDCs were isolated from male C57BL/6 mice according to a previously described protocol [15]. The cells were grown in RPMI-1640 containing 20 ng/mL M-CSF- and 10 ng/mL IL-4 under 5% CO_2_ at 37°C. On the 6^th^ day, BMDCs were treated with ART for 12 h, followed by LPS (100 ng/mL) treatment for 12 h to induce maturation. Notably, each BMDC extraction required three mice and a total of 27 C57BL/6 mice were used to extract BMDCs.

### 2.5. Heart Transplantation

Donor hearts were harvested from BALB/c mice and transplanted into the neck of recipient mice by anastomosing the graft's aorta and pulmonary artery to the recipient's neck arteries and veins. Daily cervical palpation was used to monitor graft survival, and graft rejection was defined as cessation of palpable heartbeats and verified by cervical skin discussion [16]. The partial recipients were sacrificed after the heart stopped beating to obtain the grafts and spleens. Next, the spleen was subjected to cell flow cytometry in time to screen immune cells, while the heart tissue was fixed with paraformaldehyde to prepare tissue sections. Mice that died within 24 h after modeling were considered as modeling failure. In total, there were 29 BALB/c heart donor mice and 24 mice were successfully modeled.

### 2.6. Hematoxylin-Eosin (HE) Staining

The heart grafts were harvested, formalin fixed, and paraffin embedded. Tissue blocks were sectioned into 5 *μ*m thick sections. Next, slides were baked at 60°C for 1 h, followed by deparaffinization, rehydration, and staining with hematoxylin and eosin (HE) stain. Finally, sections were evaluated by light microscopy. ImageJ software (NIH, Bethesda, MD) was then used to calculate the number of nuclei in each high-power field of view, which was used to determine the degree of infiltration of inflammatory cells.

### 2.7. Immunohistochemistry

To evaluate the expression of FOXP3, spleen sections were incubated with mouse anti-FOXP3 polyclonal antibody overnight at 4°C. Sections were then incubated with goat anti-rabbit secondary antibody for and counterstained with DAB chromogenic solution. Brown positive signals were observed under an optical microscope (BX51, Olympus Japan). ImageJ software was used to calculate the number of positive cells in each high-power field of view, which were used to evaluate the content of Tregs.

### 2.8. Immunofluorescence

To characterize the content of CD4+ and CD8+ T cells in grafts, tissue sections were incubated with primary antibodies against CD4/8 overnight at 4°C. Next, sections were incubated with Cy3-labeled goat anti-rabbit IgG (1 : 200, Beyotime, Shanghai, China) secondary antibody for 1 h at 37°C in the dark. Nuclei were counterstained with DAPI (Sigma-Aldrich, St. Louis, MO, USA). Fluorescent images were acquired using a fully automated microscope (BX63, Olympus Japan). Finally, ImageJ software was used to calculate the mean fluorescence intensity of each fluorescence picture.

### 2.9. Western Blot Analysis

To analyze the protein expression of DCs after various treatments, RIPA lysis buffer containing a mixture of protease inhibitors and phenylmethanesulfonyl fluoride (PMSF) was used to extract total protein from DCs. Protein concentration was quantified using a BCA analysis kit. Next, equal amounts of protein samples were resolved by 8-12% SDS-PAGE and then transferred to a polyvinylidene fluoride (PVDF) membrane. After blocking with PBS containing 5% skim milk, the membranes were incubated with the primary detection antibody overnight at 4°C. Next, membranes were washed with PBS and incubated with the secondary antibody. Finally, the Odyssey infrared imager was used to observe the positive binding, and relevant software (ImageJ 8.0, USA) was used to calculate the gray value of each band.

### 2.10. Flow Cytometry

For the *in vivo* assays, spleen and lymph node samples from each mouse group were harvested and prepared as single-cell suspensions by grinding. ACK lysis buffer was used to remove erythrocytes, and the remaining cells were resuspended in PBS.

For cell surface staining, the single-cell suspensions were incubated with antibody cocktails (CD4 (FITC), CD8*α* (PE), CD11c (PE), CD86 (FITC), and MHC-II (FITC)) at 37°C for 30 min.

For Tregs, the single-cell suspensions were incubated with antibody cocktails (CD4 (FITC) and CD25 (APC)) at 4°C for 30 min. Fixation/permeabilization staining (eBioscience, San Diego, CA, USA) was performed according to the manufacturer's instructions. Anti-FOXP3-phycoerythrin (PE) antibodies were used to stain the cells at 4°C for 30 min. The cells were washed twice and then the proportion of CD4+CD25+FOXP3+ cells was expressed as a percentage of the Tregs.

### 2.11. Assessment of ROS Production in DCs

To assess the effects of ART on ROS concentrations in DCs, DCs were incubated with ART and LPS for 12 h, followed by assessment of ROS concentrations with dihydroethidium (DHE) probe using flow cytometry. Changes in fluorescence intensity (FI) emitted by DHE were measured in the control, with DCs initially analyzed without DHE (blank), to ensure that there was no interference of DHE-emitted fluorescence.

### 2.12. Statistical Analysis

All statistical analyses were performed using GraphPad Prism 6.0 (GraphPad Software, USA), and the results are presented as the mean ± standard deviation (SD). Graft survival was compared using the log-rank test. For ex vivo experiments, the analysis of variance (one-way ANOVA) followed by Dunnett's test was used to compare differences among multiple groups. Other measurements were performed using unpaired Student's *t*-test. *P* values less than 0.05 were considered statistically significant.

## 3. Results

### 3.1. ART Significantly Reduces Rejection and Improves the Survival Rate of Grafts

**Figure 1:**
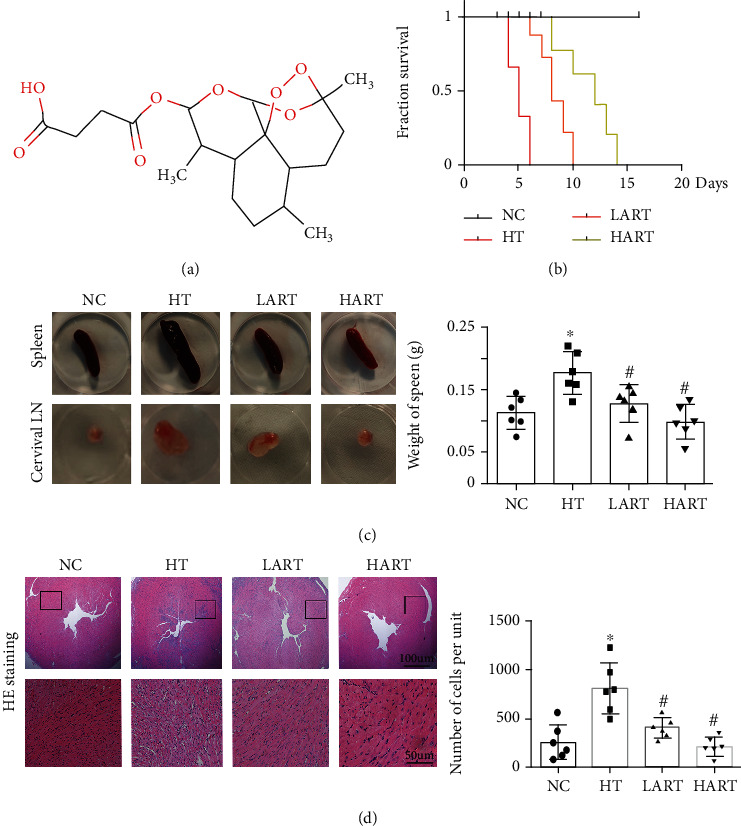
(a) Chemical molecule structure diagram of ART; (b) survival curve of grafts (*n* = 6); (c) the appearance of cervical lymph nodes and spleen in different groups; (d) weight of spleens from different groups; (e) HE staining of heart tissue and statistics graph of cells' number per unit (^∗^*P* < 0.05 compared with NC; ^#^*P* < 0.05 compared with HT).

Figure 1(a) shows the chemical structure of artesunate. In this study, a survival curve of grafts after heart transplantation was generated (Figure 1(b)). Results showed that ART treatment significantly improved the survival rate of grafts after heart transplantation, and HART (50 mg/kg) had a better effect compared to LART (10 mg/kg). Next, the spleens were isolated from recipient mice and weighed. The results indicated that the weight of spleens obtained from mice in LART and HART groups was less than the weight of those from HT group mice (Figures 1(c) and 1(d)). The reduction in the weight of spleens obtained from ART treatment mice can indirectly indicate the reduction of immune rejection. After HE staining of heart tissue sections, it was found that ART significantly reduced the infiltration of inflammatory cells (Figure 1(e)). Moreover, HE staining proved that ART maintained the survival of the grafts and reduced the rejection of heart transplantation.

### 3.2. ART Reduces the Immune Cell Infiltration and Improves Immune Environment after Heart Transplantation

**Figure 2:**
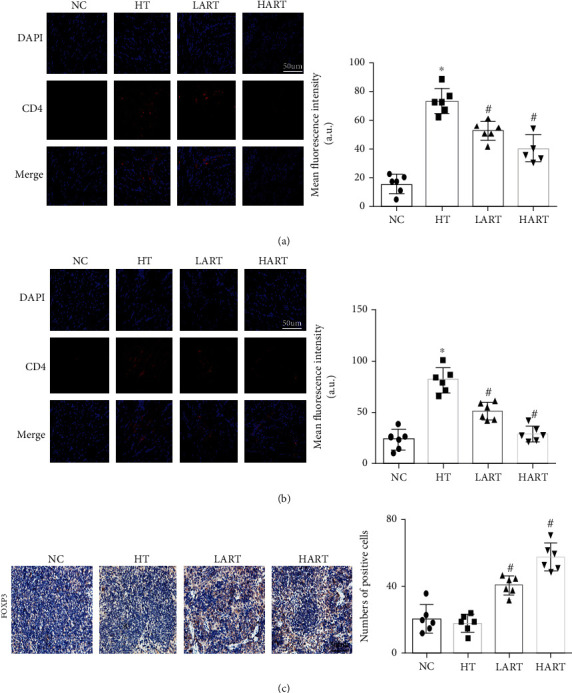
(a) Immunofluorescence staining and mean fluorescence intensity of CD4 in grafts (blue: DAPI, red: CD4); (b) immunofluorescence staining and mean fluorescence intensity of CD8 in grafts (blue: DAPI, red: CD8); (c) immunohistochemistry of FOXP3 and statistics graph of positive cells' number per unit (^∗^*P* < 0.05 compared with NC; ^#^*P* < 0.05 compared with HT).

**Figure 3:**
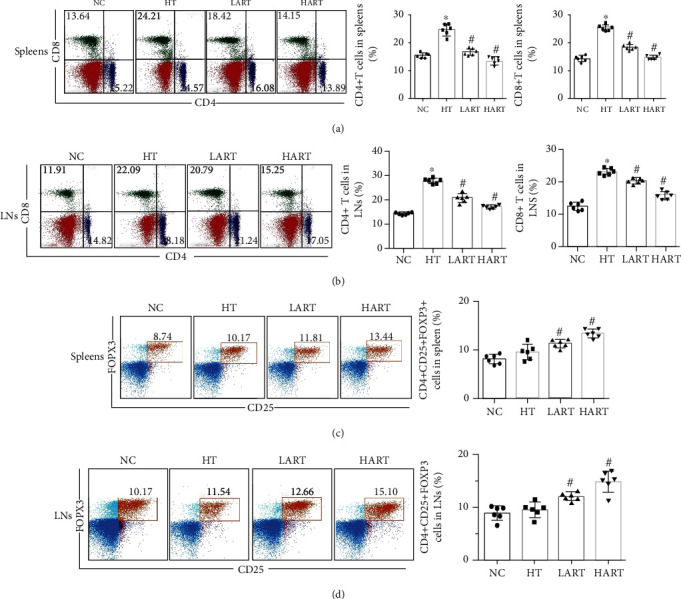
(a) Flow cytometric sorting of CD4+/8+ T cells in spleens and statistics chart; (b) flow cytometric sorting of CD4+/8+ T cells in LNs and statistics chart; (c) CD4+CD25+FOXP3+ cells in spleens and statistics chart; (d) CD4+CD25+FOXP3+ cells in LNs and statistics chart (^∗^*P* < 0.05 compared with NC; ^#^*P* < 0.05 compared with HT).

Sections of heart tissues obtained from HT mice indicated that ART treatment can reduce the CD4+ and CD8+ T cell infiltration in allogeneic grafts (Figures 2(a) and 2(b)). Immunohistochemistry results showed that the positive rate of FOXP3 (a specific marker of regulatory T cells (Tregs)) in the spleens increased significantly after ART treatment (Figure 2(c)). After cell flow sorting, it was found that ART reduced the content of CD4+ and CD8+ T cells in the spleen and lymph nodes (LNs) of the recipients (Figures 3(a) and 3(b)) and, at the same time, increased Tregs. It is worth noting that the main role of Tregs is to maintain self-tolerance and avoid excessive immune response to damage of the body. In addition, the protective effect of ART increased with the increased dose of ART (Figures 3(c) and 3(d)). These results suggest that ART can reduce the immune cell infiltration and improve the immune environment after transplantation.

### 3.3. ART Alleviates Oxidative Stress and Inhibits Maturation of DCs

**Figure 4:**
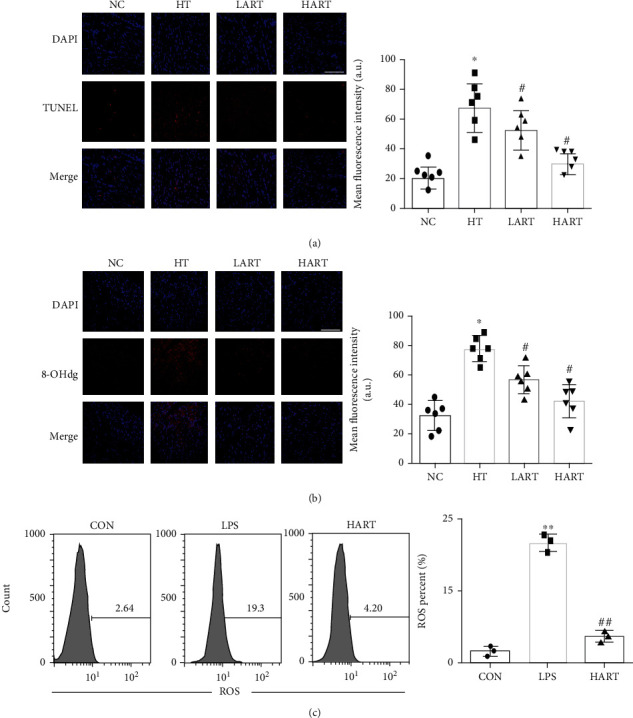
(a) TUNEL staining and mean fluorescence intensity of the heart (blue: DAPI, red: TUNEL); (b) 8-OHdg staining mean fluorescence intensity of the heart (blue: DAPI, red: 8-OHdg; ^∗^*P* < 0.05 compared with NC; ^#^*P* < 0.05 compared with HT); (c) ROS of DCs after stimulation with LPS (^∗∗^*P* < 0.01 compared with CON; ^##^*P* < 0.01 compared with LPS).

TUNEL staining results revealed that ART effectively reduced cardiomyocyte apoptosis in allograft hearts (Figure 4(a)). The immunofluorescence of grafts showed that ART also reduced the expression of 8-OHdg (a biomarker of DNA oxidative damage) in the grafts (Figure 4(b)). This suggests that ART can effectively reduce oxidative stress damage during heart transplantation, and this protective effect is enhanced in a dose-dependent manner. After LPS stimulation, it was found that ART can effectively reduce production of ROS in DCs and then place DCs in an immature state (Figure 4(c)). Collectively, these results suggest that ART can inhibit maturation of DCs by reducing the oxidative stress damage of the transplanted heart.

### 3.4. ART Inhibits Maturation of DCs by Activating the PERK/ATF4/CHOP Signaling Pathway

**Figure 5:**
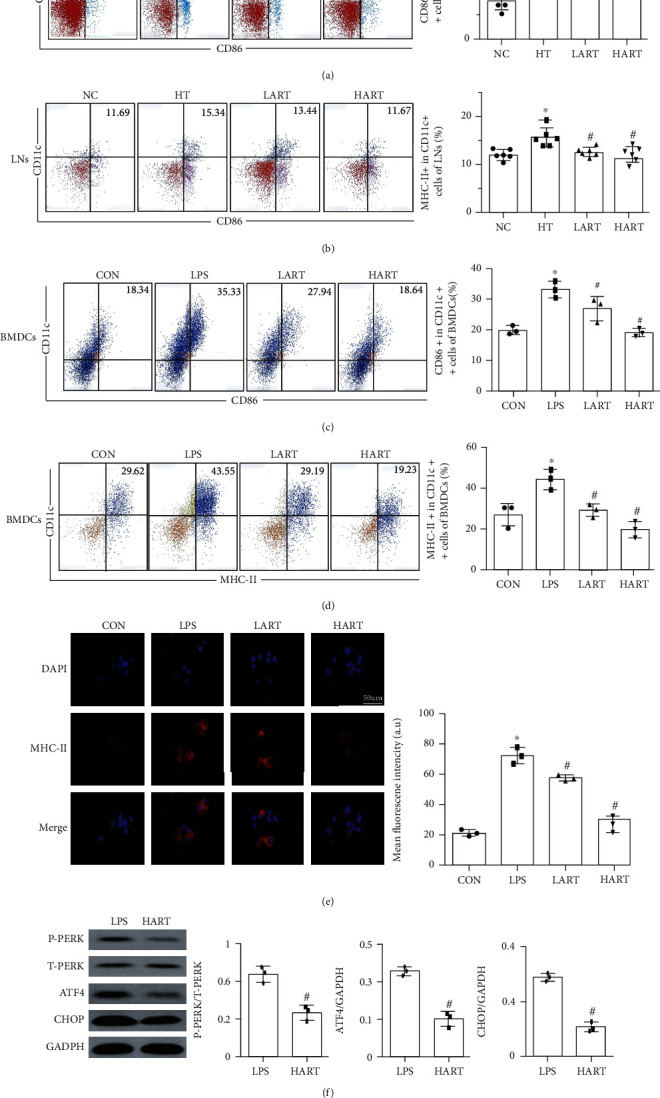
(a) Flow cytometric sorting of CD11c+ and CD86+ cells in lymph nodes; (b) flow cytometric sorting of CD11c+ and MHC-II+ cells in lymph nodes (^∗^*P* < 0.05 compared with NC; ^#^*P* < 0.05 compared with HT); (c) flow cytometric sorting of CD11c+ and CD86+ cells in BMDCs; (d) flow cytometric sorting of CD11c+ and MHC-II+ cells in BMDCs (^∗^*P* < 0.05 compared with CON; ^#^*P* < 0.05 compared with LPS); (e) immunofluorescence staining and mean fluorescence intensity of MHC-II in BMDCs (blue: DAPI, red: MHC-II); (f) western blotting of PERK/ATF4/CHOP signaling pathway (^#^*P* < 0.05 compared with LPS).

CD11c, CD86, and MHC-II (surface markers of DC maturation) were detected in spleens and lymph nodes of recipients. Results showed that ART treatment can reduce the expression of CD86 and MHC-II, the surface maturity markers of DCs (Figures 5(a) and 5(b)). It was also found that ART significantly inhibited maturation of BMDCs *in vitro* (Figures 5(c)–5(e)). Western blot results showed that the expression of p-PERK/ATF4/CHOP proteins was significantly decreased in LART and HART groups, indicating that ER stress was suppressed by ART treatment (Figure 5(f)). Therefore, it was evident that ART can inhibit the development of immature dendritic cells (imDCs) into mature dendritic cells (mDCs) by restraining the PERK/ATF4/CHOP signaling pathway.

### 3.5. ER Signaling Pathway Is Essential for ART to Suppress the Maturation of DCs

**Figure 6:**
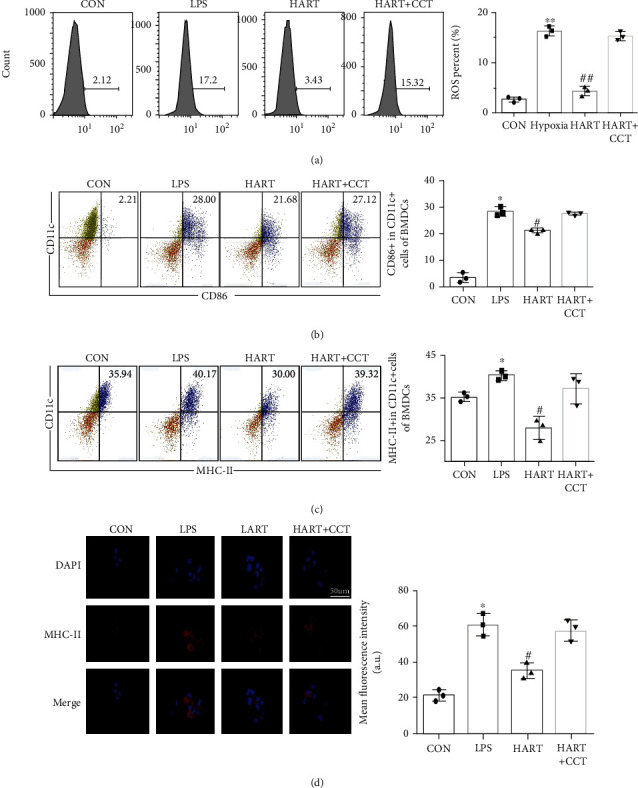
(a) ROS of DCs after LPS and CCT (^∗∗^*P* < 0.01 compared with CON; ^##^*P* < 0.01 compared with LPS); (b) flow cytometric sorting of CD11c+ and CD86+ cells in BMDCs; (c) flow cytometric sorting of CD11c+ and MHC-II+ cells in BMDCs (^∗^*P* < 0.05 compared with CON; ^#^*P* < 0.05 compared with LPS); (d) immunofluorescence staining and mean fluorescence intensity of MHC-II in BMDCs (blue: DAPI, red: MHC-II).

**Figure 7:**
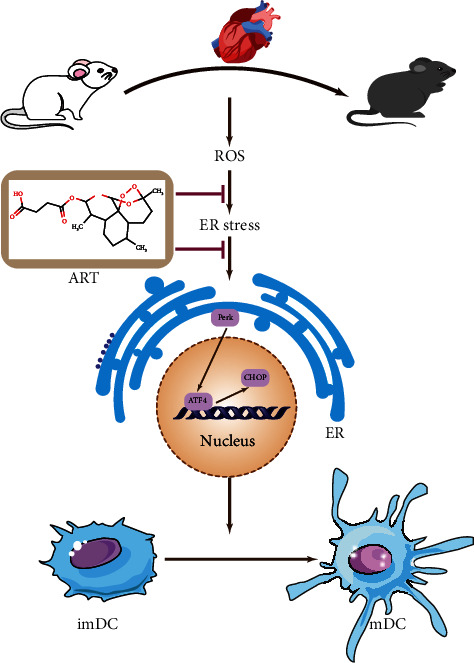
The mechanism of ART in BMDCs. ART reduces the generation of ROS to alleviate the endoplasmic reticulum stress and inhibits the maturation of DCs via reducing the expression of the PERK/ATF4/CHOP protein.

Furthermore, CCT020312 (CCT, a novel specific PERK activator) was used to treat DCs, and it was found that the positive effect of ART was partially reversed by CCT administration. In addition, the production of ROS in DCs was reactivated (Figure 6(a)), and the immature state of DCs could not be maintained (Figures 6(b)–6(d)). These results proved that the ER signaling pathway is a crucial pathway for the protective role of ART.  Figure 7 shows the mechanism of ART in BMDCs.

## 4. Discussion

Acute rejection has always been the main factor that limits the development of heart transplantation, and is also an important cause of surgical failure and patient death. Although immunosuppressants are normally used to treat rejection in the clinic, they may lead to deleterious side effects after long-term use. Therefore, many researchers have focused on natural compounds extracted from plants as immunosuppressants. Evidence suggests that ART may be an ideal drug to alleviate immune rejection because of its good anti-inflammatory and anti-immune effects [17, 18]. The results of this study showed that ART can improve the survival rate of grafts after HT, reduce the infiltration of immune cells (CD4+/8+ T cells), and regulate immune tolerance via enhancing the scale of Tregs. At the same time, ART inhibits maturation of BMDCs through mitigating ER stress. Notably, the ameliorating effect of ART was reversed after administration of CCT020312 (CCT, a novel specific PERK activator). Collectively, these results suggest that ART plays a critical role in HT through the PERK/ATF4/CHOP pathway.

In recent years, the widening gap between the number of waiting recipients and the number of donors has resulted in a continuing trend toward transplanting urgent status recipients [1]. However, the development of heart transplantation is not only limited by the insufficient number of donors but is also mainly affected by many complications [19, 20]. Early complications of heart transplantation include graft dysfunction, acute rejection, infection, and renal function injury. On the other hand, the long-term common complications are mainly malignant tumor and cardiac allograft vasculopathy (CAV). The occurrence and development of these complications is closely associated with the immune system and the functional transformation of some immune cells [21].

When an allograft heart is introduced to the recipient, it is recognized by the DCs of the immune system, absorbed and processed, and then presented to T cells [22]. Activation of T lymphocytes modulates the adaptive immune responses that drive innate immunity and adaptive immunity. Under the stimulation of an antigen, DCs will change from the immature state to the mature state, thereby playing the function of presenting the antigen [23]. Therefore, inhibiting the development of DCs can effectively inhibit the occurrence of acute rejection [24]. In this study, the content of CD4+ and CD8+ T cells in the heart and surrounding lymphatic tissues (spleens and lymph nodes) was determined to express the severity of immune rejection. Regulatory T cells (Tregs), a subgroup of T cells that control autoimmune reactivity in the body, can reduce the rejection of heart transplantation. Results obtained in this study showed that ART treatment can effectively reduce the content of CD4+ and CD8+ T cells in recipient mice and also increase the proportion of Tregs which protect the graft [25, 26]. The cell experiments also indicated that ART treatment can effectively reduce the expression of MHC-II and CD86, the surface markers of DC maturation, in the BMDCs. Therefore, we concluded that ART can protect the graft by inhibiting maturation of DCs.

Previous studies have reported that production of excessive ROS enhances the apoptosis of cardiomyocytes and activates endoplasmic reticulum (ER) stress [27, 28]. The ER is a multifunctional organelle that regulates a variety of physiological processes, and most of the intracellular protein synthesis occurs in its lumen. Destruction of ER protein folding ability leads to the accumulation of unfolded and misfolded proteins, with the resulting disturbance of ER homeostasis being called ER stress [29, 30]. To counteract the elevated ER stress, adaptive mechanisms, including ER-associated degradation (ERAD), the unfolded protein response (UPR), and reticulophagy, are activated. The UPR attempts to restore proteostasis, but persistent UPR activation can lead to a maladaptive response. The signal is mediated by three different stress sensors located on the ER membrane, including inositol-requiring enzyme 1(IRE1), double-stranded RNA-activated protein kinase-like ER kinase (PERK), and activating transcription factor 6 (ATF6) [31–33]. Among the three, the PERK/eIF2*α* pathway is the most closely associated with oxidative stress. Several studies have shown that ROS may not normally act upstream of ER stress, but it clearly activates certain parts of UPR. Exogenous H_2_O_2_ or organic oxidants, such as tert-butyl hydroperoxide, menadione, or diamide, can cause phosphorylation of eIF2*α*, induce ATF4, and mediate an integrated stress response. Interestingly, it is not clear whether PERK or any other upstream kinase is involved in this phosphorylation event. The downstream targets of PERK/eIF2*α* and ATF4 include antioxidant-related genes. In addition, PERK activation causes the antioxidant transcription factor Nrf2 to dissociate from its inhibitor Keap1, thereby increasing the level of intracellular glutathione. When ERS occurs, PERK can form oligomers through autophosphorylation, thereby promoting the phosphorylation of downstream eukaryotic translation initiation factor 2*α* (eIF2*α*). This has an overall effect of reducing cell pressure and inhibiting protein synthesis to keep cells alive. However, excessive or persistent ERS may skip phosphorylation of eIF2*α* and activate PERK's downstream transcription factor-activated transcription factor 4 (ATF4). Consequently, the activated ATF4 can increase the transcription and expression levels of CHOP. It is well known that CHOP is a key proapoptotic molecule that can cause personal injury by preventing the cell cycle and inducing cell death [34]. Overall, these studies show that ROS is clearly located upstream of a specific part of UPR and may play a role in the physiological function between ER stress and UPR.

How does ART inhibit the maturation of DCs and the generation of ROS? ART is a widely studied artemisinin derivative that can be converted to dihydroartemisinin (DHA), the active metabolite, after entering the human body [35–37]. ART has good water solubility and higher oral bioavailability because of its hemisuccinate groups, which makes it have more advantageous pharmacological characteristics [38, 39]. This study has shown that administration of ART in HT mice reduces the occurrence of acute rejection and the oxidative stress damage. We found that ART reduces ER stress by reducing the ROS-mediated production of DCs, thereby maintaining the immature state of DCs [40, 41]. Therefore, the antigen presentation function of DC is reduced, and ultimately, the immune rejection after HT is reduced. Moreover, ART inhibits the apoptosis of cardiomyocytes by reducing the generation of ROS. Results obtained in this study showed that ART can reduce the phosphorylation of PERK and the expression of ATF4 and CHOP. After administration of a specific PERK activator (CCT) [42, 43], it was found that the protective effect of ART was reduced, and thus, the maturation of DCs could not be inhibited. This suggests that ART affects the development and maturation of DCs through the PERK/ATF4/CHOP signal pathway.

However, this study had some limitations. Firstly, the study only focused on the role and mechanism of ART in acute rejection of heart transplantation and did not determine the long-term prognosis. In our future experiments, we will construct a mouse model of chronic rejection of heart transplantation to further study the role of ART. Secondly, the results showed that ART reduced the expression of 8-OHDG and TUNEL after heart transplantation. However, these results do not clearly indicate whether oxidative stress damage was caused by ischemia-reperfusion (IR) or immune rejection. Therefore, further studies are required to determine whether ART alleviates oxidative stress injury by alleviating IR or immune rejection. Thirdly, to elucidate the underlying mechanism, we only studied expression at the protein level rather than at both the gene and protein levels due to time and funding constraints. Therefore, our future studies will incorporate molecular hybridization to identify ART targets, which will provide better results for application in clinical practice [44].

## 5. Conclusion

This study reports, for the first time, that ART regulates maturation of dendritic cells and acute rejection in mice after HT. The results provide supporting evidence that ART may inactivate the PERK/ATF4/CHOP signaling pathway, thereby inhibiting the maturation of dendritic cells. The findings will help to reveal new treatment strategies for heart transplantation. Moreover, *in vivo* and *in vitro* experiments were conducted to determine the protective effect and mechanism of ART in the early stage of heart transplantation, with results proving that ART may be a potential immunosuppressant. Notably, ART is an artemisinin derivative, and its biosecurity has been proven in clinical application. Collectively, the findings of this study suggest that ART is a novel potential therapeutic drug for preventing acute rejection after heart transplantation [45].

## Data Availability

The original data used to support the findings of this study are available from the corresponding author upon request.
